# Impact of body mass index and waist-to-hip ratio on mortality in middle-aged Koreans: a prospective cohort study based on a Health Examinees study

**DOI:** 10.4178/epih.e2024073

**Published:** 2024-09-02

**Authors:** Sooyoung Cho, Aesun Shin, Ji-Yeob Choi, Jong-Koo Lee, Daehee Kang

**Affiliations:** 1 Genomic Medicine Institute, Medical Research Center, Seoul National University College of Medicine, Seoul, Korea; 2 Department of Preventive Medicine, Seoul National University College of Medicine, Seoul, Korea; 3 Integrated Major in Innovative Medical Science, Seoul National University College of Medicine, Seoul, Korea; 4 Cancer Research Institute, Seoul National University, Seoul, Korea; 5 Department of Biomedical Science, Seoul National University Graduate School, Seoul, Korea; 6 Department of Family Medicine, Seoul National University Hospital, Seoul, Korea

**Keywords:** Body mass index, Waist-hip ratio, Obesity, Mortality

## Abstract

**OBJECTIVES:**

We aimed to assess the impact of obesity on mortality in middle-aged Koreans using data from a Health Examinees study.

**METHODS:**

We used data from the participants who had complete information on body size and gave informed consent for the linkage of their data with the national death certificate data. Cox proportional hazard model was used to estimate the hazard ratios and 95% confidence intervals (CIs) of body mass index (BMI) and waist-to-hip ratio (WHR) for all-cause, cardiovascular, and cancer mortality.

**RESULTS:**

A total of 115,961 participants were included in the study. The results showed a U-shaped association between BMI and mortality, indicating that both males and females with BMIs of less than 21.0 kg/m^2^ and greater than or equal to 30.0 kg/m^2^ are at increased risk. The results showed that males with a BMI of less than 18.5 kg/m² had a significantly higher risk of all-cause mortality (adjusted hazard ratio [aHR], 2.24; 95% CI, 1.73 to 2.91) and cardiovascular mortality (aHR, 2.27; 95% CI, 1.23 to 4.20). Similarly, males with a WHR of less than 0.80 (aHR, 1.38; 95% CI, 1.08 to 1.77), 0.90 to less than 0.95 (aHR, 1.15; 95% CI, 1.02 to 1.29), and greater than or equal to 0.95 (aHR, 1.28; 95% CI, 1.11 to 1.47) showed an increased risk of all-cause mortality. In females, a BMI of less than 18.0 kg/m^2^ was linked to a higher risk of cardiovascular mortality (aHR, 2.67; 95% CI, 1.13 to 6.33).

**CONCLUSIONS:**

Being underweight was associated with an increased risk of mortality in both sexes, and the lowest risk of death was found in males who were slightly overweight with a BMI of 23.0-25.0 kg/m^2^.

## INTRODUCTION

Obesity is the major cause of chronic diseases and the leading cause of mortality. Global obesity prevalence has increased significantly and tripled from 1975 to 2016 [1]. A similar trend has been observed in Korea, with an increase in obesity prevalence from 29.7% in 2009 to 36.3% in 2019 [2]. People with obesity are at increased risk of cardiovascular diseases, type 2 diabetes, certain cancers, and premature death [3,4]. The Global Burden of Disease Study reported that obesity accounted for 5.02 million deaths and 160 million disability-adjusted life-years worldwide in 2019 [5]. As obesity has emerged, the burden of disease attributed to this condition increases accordingly.

Body mass index (BMI) is a widely used anthropometric measure of general obesity. A J-shape association between BMI and mortality has been suggested by previous meta-analyses [6,7]. This J-shape relationship may vary by geographical region, with a weaker association observed in East Asian populations than in North American and European populations [7]. Considering that adverse health outcomes are associated with a lower BMI than that recommended by the World Health Organization (WHO) in Western countries, the WHO recommended a lower BMI cut-off point for Asian populations [8].

Abdominal obesity has also become a global public health concern, as the prevalence of obesity is steadily increasing around the world. The findings, from a systematic review and meta-analysis of data from more than 19 million participants in 186 countries, show a significant global increase in the prevalence of abdominal obesity since 1980, with an estimated 20% of adults worldwide having abdominal obesity in 2015 [9]. The prevalence of abdominal obesity in Korea has also shown an increasing trend in recent years. According to the Korea National Health and Nutrition Examination Survey (KNHANES), the prevalence of abdominal obesity has increased steadily from 19.0% in 2009 to 23.9% in 2019 [2]. The relationship between waist circumference and health outcomes may be influenced by ethnic differences in body composition, fat distribution and genetic factors [10].

As a result, using the same cut-offs of obesity for different populations may lead to misclassification of individuals, either underestimating or overestimating their risk of developing obesity-related health conditions. In this study, we aimed to evaluate the impact of BMI and waist-to-hip ratio (WHR) on cause-specific mortality in a Health Examinees (HEXA) study.

## MATERIALS AND METHODS

The HEXA study is conducted as a part of a policy research project called Korea Genome Epidemiology Study (KoGES), which is supported by the National Institutes of Health of Korea [11], and is also referred to as KoGES_HEXA. Participants in the HEXA study were prospectively enrolled from 2004 to 2013 at 38 health examination centers and training hospitals located in eight regions based on the national health checkup services funded by the Korean Centers for Disease Control and Prevention [11].

We included 173,195 HEXA participants in the analyses, specifically Health Examinees-Gem (HEXA-G) participants. The criteria for selecting HEXA-G participants have been described earlier [12]. We set up the study participants separately for BMI and WHR to minimize the number of excluded participants. A total of 139,263 HEXA-G participants were included, of whom, we excluded participants who were missing information on anthropometric measurements of height and weight, and waist and hip circumference for constructing each dataset for the analyses of BMI and WHR. And we additionally excluded participants who did not provide a personal identification number or did not consent to linking their data with the national death certificate data or providing the linked data to a third. The participants with the same year and month of enrollment and death were also excluded. Finally, 115,961 and 115,504 participants were included in the analyses of BMI and WHR, respectively.

Training interviewers interviewed participants, who responded to a structured questionnaire on general characteristics and past medical history. Anthropometric measurements were also conducted for all participants. Height was measured using digital freestanding stadiometers (BSM270/BSM330; InBody Co., Seoul, Korea) with the participant’s head in the Frankfort horizontal plane and was read up to one decimal place. Weight was measured using digital scales (BSM270/BSM330; InBody Co.) in units of 10 g. BMI was calculated by dividing weight in kilograms by the square of height in meters. We categorized participants into seven groups based on their BMI as follows: < 18.5, 18.5-< 21.0, 21.0-< 23.0, 23.0-< 25.0, 25.0-< 27.0, 27.0-< 30.0, and ≥ 30.0 kg/m^2^. Mortality was ascertained via the linking of patients’ data to the death certificated database of Statistics Korea until December 31, 2021. We defined all-cause mortality as all-cause mortality, cancer mortality as the C code, and cardiovascular mortality as the I code using the International Classification of Diseases 10th revision. We specifically defined all-cause deaths as excluding codes V-Y (external causes of morbidity) and Z (factors influencing health status and contact with health services) because of the limited explanation of deaths from external causes by obesity. A total of 3,050 deaths were observed in the study population for BMI with a median follow-up year of 10.13 years, and 3,051 deaths were observed in the study population for WHR with a median follow-up year of 10.13 years.

Cox proportional hazard model was used for estimating hazard ratios (HRs) and 95% confidence intervals (CIs) of BMI and WHR on cause-specific mortality with age as the time scale. We adjusted for years of education and habits of smoking, alcohol consumption, and regular physical activity, and additionally for menopausal status among females. All statistical analyses were stratified by sex. R version 4.1.2 (R Foundation for Statistical Computing, Vienna, Austria) were used for constructing data and conducting statistical analyses.

### Ethics statement

The Institutional Review Board of Seoul National University Hospital (No. 2212-006-1381) approved the study protocol.

## RESULTS

Table 1 shows the participants’ demographic and lifestyle characteristics in the study of BMI and mortality, stratified by sex. A total of 115,961 participants were included, with 39,689 males and 76,272 females. The mean at enrollment was 53.7 years with a standard deviation (SD) of 8.4 for males and 52.4 years with a SD of 7.7 for females. Most participants had at least 12 years of education, with 40.6% of males and 43.2% of females having 12-16 years of education and 37.7% of males and 20.1% of females having ≥16 years of education. Males were more frequently former (41.1%) or current smokers (31.3%) than females (1.3 and 2.3%, respectively). Most males were current drinkers (72.5%), whereas 66.7% of females reported never drinking; more than half of both males (57.4%) and females (51.1%) exercised regularly. Approximately, 59.3% of females were categorized as postmenopausal.

Table 2 shows the demographic characteristics of males and females in the study of WHR and mortality. The total number of males and females in the study included 39,522 and 75,982 respectively. Males have a slightly higher mean age of 53.7=8.7 years, while females have a mean age of 52.4=7.7 years. The age range 50-59 is the most common for both sexes. Regarding the educational level, males have a higher percentage of those with 16 or more years of education than females. A larger proportion of males have been smokers and drinkers, whereas most females were never smokers and consume less alcohol. In addition, a slightly higher proportion of males than females reported regular physical activity. Among females, 59.3% of people reported having undergone the menopause.

Table 3 shows the HRs (95% CIs) of BMI on cause-specific mortality among males and females, compared to those with a BMI of 21.0-< 23.0 kg/m^2^. Males with a BMI of < 18.5 kg/m^2^ had increased risks of all-cause mortality (adjusted hazard ratio [aHR], 2.24; 95% CI, 1.73 to 2.91) and cardiovascular mortality (aHR, 2.27; 95% CI, 1.23 to 4.20). A BMI of 18.5-< 21.0 kg/m^2^ was associated with an increased risk of all-cause mortality with marginal significance (aHR, 1.18; 95% CI, 1.00 to 1.39). We also found decreased risks of all-cause mortality among males with BMIs of 23.0-< 25.0 kg/m^2^ (aHR, 0.85; 95% CI, 0.74 to 0.97) and 25.0-< 27.0 kg/m^2^ with marginal significance (aHR, 0.87; 95% CI, 0.75 to 1.00). Males with a BMI of ≥ 30.0 kg/m^2^ were associated with an increased risk of cancer mortality. Among females, a BMI of < 18.0 kg/m^2^ was associated with cardiovascular mortality (aHR, 2.67; 95% CI, 1.13 to 6.33), but the range of CIs was wide because of the limited number of cases. Females with a BMI of 18.5-< 21.0 kg/m^2^ had an increased risk of all-cause mortality (aHR, 1.23; 95% CI, 1.01 to 1.50). We found a significant association between a BMI of 27.0-< 30.0 kg/m^2^ and an increased risk of cardiovascular mortality, but the statistical significance became marginal after adjustment (HR, 1.73; 95% CI, 1.09 to 2.73; aHR, 1.56; 95% CI, 0.99 to 2.48). Similarly, a BMI of ≥ 30.0 kg/m^2^ was also associated with an increased risk of all-cause mortality, although this statistical significance became marginal after adjustment (HR, 1.42; 95% CI, 1.06 to 1.90; aHR, 1.33; 95% CI, 0.99 to 1.78).

Table 4 presents the HRs and corresponding 95% CIs of the association between WHR and cause-specific mortality among males and females, with comparison to those with the WHR of 0.85-< 0.90. We found that males with the WHR of < 0.80 (aHR, 1.38; 95% CI, 1.08 to 1.77), 0.90-< 0.95 (aHR, 1.15; 95% CI, 1.02 to 1.29), and ≥ 0.95 (aHR, 1.28; 95% CI, 1.11 to 1.47) was significantly associated with the increased all-cause mortality. And we observed similar pattern for cardiovascular mortality only among males with the WHR of ≥ 0.95 (aHR, 1.48; 95% CI, 1.08 to 2.02) show elevated cardiovascular mortality. In contrast, for cancer mortality, we cannot find any significant association. Among females, both lower and higher WHR did not significantly impact to all-cause or cancer mortality. We observed elevated all-cause mortality among females with a WHR of ≥ 0.95 (HR, 1.26; 95% CI, 1.01 to 1.58), but it did not reach the level of significance after the adjustment (aHR, 1.21; 95% CI, 0.97 to 1.51).

**Figure 1.**
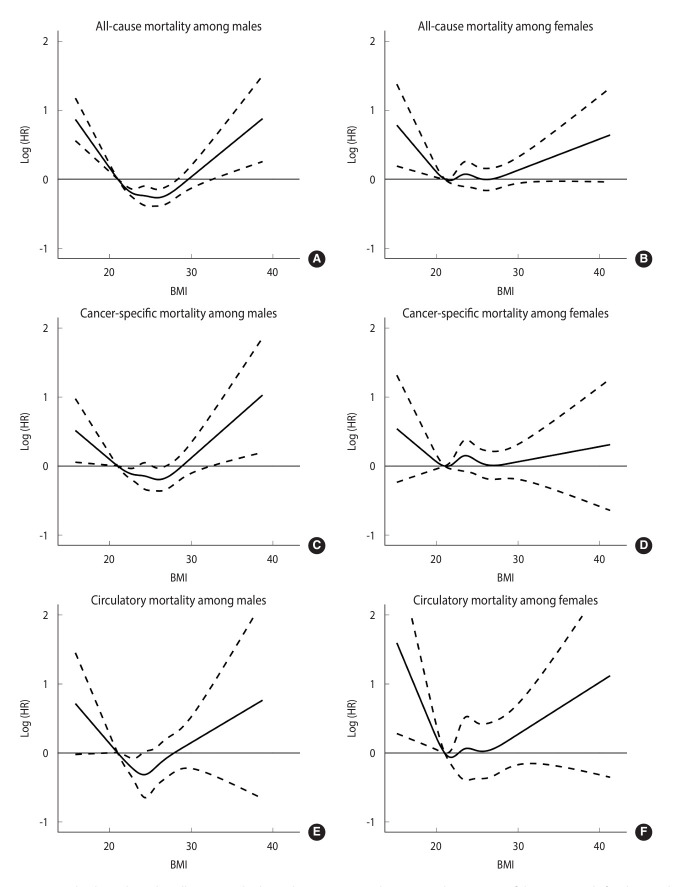
Restricted cubic spline plots illustrating the hazard ratios (HRs) and corresponding 95% confidence intervals for the non-linear association between the body mass index (BMI) and cancer mortality among males and females, compared to the BMI of 21.0 kg/m^2^. All-cause mortality (A: male, B: female). Cancer-specific mortality (C: male, D: female). Circulatory mortality (E: male, F: female). The age at recruitment was used as the time scale. Years of education (<12, 12-16, and ≥16 years) and habits of smoking, alcohol consumption (neve, former, and current), and regular physical activity (no and yes), and additionally for menopausal status among females (no and yes) were adjusted. The y-axis is on a logarithmic scale to better visualization. We fitted the restricted cubic spline function with 5 knots at the quantiles (0.050, 0.275, 0.500, 0.725, and 0.950) of BMI.

Figure 1 shows the restricted cubic spline plot demonstrating a non-linear association between BMI and all-cause mortality in both sexes, compared to those with a BMI of 21.0 kg/m^2^. Both males and females with BMIs of < 21.0 kg/m^2^ had an increased risk of all-cause mortality. The curve for males is steeper than that for females, and a stronger impact of BMI on all-cause mortality might be suggested in males than in females. The curve for males also shows a more clear U-shape than that for females. In particular, a relatively flat section is seen in the middle of the curve for females. It suggests that the risk for all-cause mortality among females could be independent of the impact of a BMI of > 21.0 kg/m^2^.

**Figure 2.**
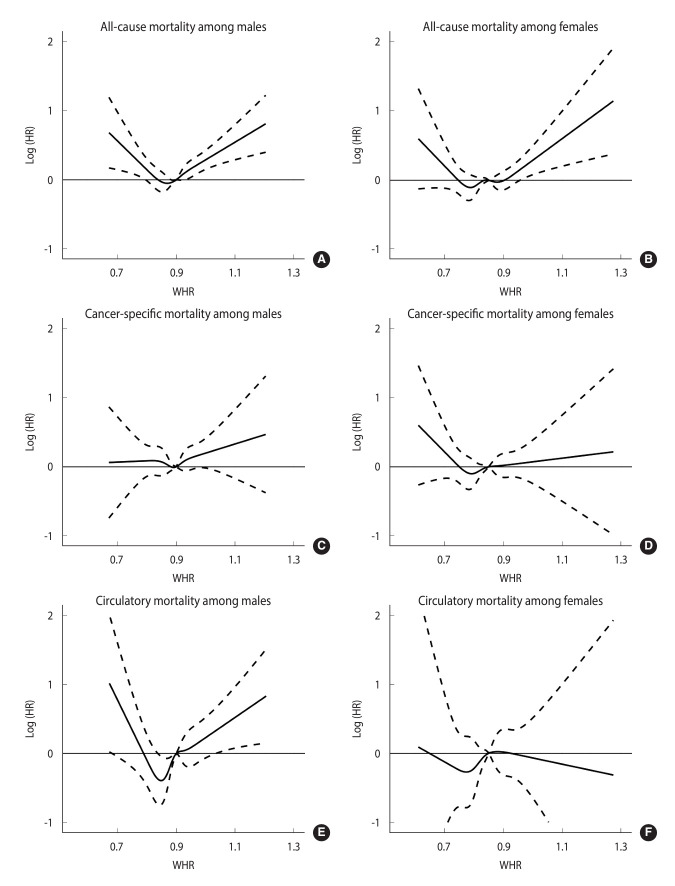
Restricted cubic spline plots illustrating the hazard ratios (HRs) and corresponding 95% confidence intervals for the non-linear association between the waist-to-hip ratio (WHR) and cause-specific mortality among males and females, compared to 0.85. All-cause mortality (A: male, B: female). Cancer-specific mortality (C: male, D: female). Circulatory mortality (E: male, F: female).The age at recruitment was used as the time scale. Years of education (<12, 12-16, and ≥16 years) and habits of smoking, alcohol consumption (never, former, and current), and regular physical activity (no and yes), and additionally for menopausal status among females (no and yes) were adjusted. The y-axis is on a logarithmic scale to better visualization. We fitted the restricted cubic spline function with 5 knots at the quantiles (0.050, 0.275, 0.500, 0.725, and 0.950) of the WHR.

Figure 2 illustrated the non-linear association between the WHR and cause-specific mortality among males and females, compared to the WHR of 0.85. For all-cause mortality, both males and females demonstrate a U-shaped association between WHR and all-cause mortality, with males showing a steeper curve. In contrast, we observed an approximately horizontal curve for the association between WHR and cancer mortality, and there was no statistically significant association with cancer mortality across all ranges of WHR. Regarding cardiovascular mortality, males showed a slight U-shaped association, whereas females showed no association.

## DISCUSSION

We evaluated the impact of BMI and WHR on cause-specific mortality in middle-aged Korean. The impact of BMI on mortality differed by sex, with increased risks observed in participants with BMIs of < 21.0 kg/m^2^ for both sexes. Notably, a BMI of 23.0-< 25.0 kg/m^2^ was linked to decreased mortality risk in males only. The non-linear association between BMI and mortality, evaluated through restricted cubic spline plots, revealed a greater impact on mortality for males than for females. In a separate investigation on WHR, mortality’s impact was more pronounced among males, showing a U-shaped association. Both lower and higher WHRs were associated with increased all-cause and cardiovascular mortality, suggesting an optimal range of 0.85-0.90 for health. Conversely, no significant association with cancer mortality was found in males. In females, WHR had no significant impact on all-cause or cancer mortality, but a lower ratio was associated with a significant reduction in cardiovascular mortality.

A previous study analyzed the pooled data from the Asia Cohort Consortium consisting of over 1 million Asian participants and reported that BMIs of < 20.0 kg/m^2^ and > 27.6 kg/m^2^ were significantly associated with the risk of all-cause mortality, compared with a BMI of 22.6-25.0 kg/m^2^ [13]. Specifically, the greatest impact on mortality was observed in the lowest BMI category (≤ 15.0 vs. 22.6-25.0 kg/m^2^: aHR, 2.76; 95% CI, 1.88 to 4.07), consistent with our findings for males (< 18.5 vs. 21.0-< 23.0 kg/m^2^: aHR, 2.24; 95% CI, 1.73 to 2.91). The association between overweight and reduced mortality has been termed as the “obesity paradox.” Slightly overweight individuals reportedly have improved insulin resistance and immune response [14], which can lead to reduced mortality. This phenomenon can also be explained by increased energy reserves in the form of adipose tissue, which can be resistant to a hypercatabolic state with increased metabolic demands [15]. The concept of “metabolically healthy obesity” addresses the paradoxical association between BMI and improved health outcomes and considers both BMI and the presence of metabolic abnormalities [16].

There have also been studies in the Korean population based on data from the National Health Insurance Service. The study evaluating the association between BMI and mortality in 1.3 million Koreans whose deaths were followed up to 2004, based on national insurance data from 1992 to 1995, reported that death from any cause had a J-shaped association with BMI in both sexes, and suggested that the association between BMI and death might be modified by age, sex, and smoking history [17]. A more recent study using Korean National Health Insurance data followed 12.8 million Korean adults who underwent a health examination between 2001 and 2004 and were followed up until 2013 to assess the association between BMI and mortality, stratified by a combination of sex, age group and smoking history [18]. The results showed that the BMI associated with the lowest mortality increased with age and was lower in females than in males.

There have also been several studies on obesity and mortality have been conducted in Korean population. A study using data from the National Sample Cohort database of the Korean National Health Insurance Service has shown the U-shaped association between all-cause mortality and has also highlighted cardiovascular deaths among those with the lowest BMI [19]. A pooled analysis of three population-based prospective cohorts in Korea, including HEXA, recently published the association between BMI and mortality [20]. This study also reported a U-shaped association between BMI and all-cause mortality, cardiovascular mortality, and cancer mortality and found no survival benefit from being overweight or obese compared with being of normal weight. Our results also showed an increased risk of cardiovascular mortality with a low BMI of < 18.5 kg/m^2^ in both males (aHR, 2.27; 95% CI, 1.23 to 4.20) and females (aHR, 2.67; 95% CI, 1.13 to 6.33), compared to a BMI of 21.0-< 23.0 kg/m^2^, although we did not find a significant association between a BMI of > 23.0 kg/m^2^ and the risk of cardiovascular mortality. Low body weight is generally associated with low muscle mass, and decreased muscle mass is known to be associated with insulin resistance; accordingly, reduced body metabolism can lead to cardiovascular diseases [21].

In a study on WHR, both lower and higher WHRs were associated with an increased mortality from all causes and from cardiovascular disease, showing a U-shaped association, suggesting that a certain range (0.85-0.90) may be optimal for health in males. Conversely, no significant association was found between WHR and mortality in females after adjustment. Our results contrast with previous meta-analyses, which found a stronger association in females than in males and a linear association between WHR and mortality [22]. This difference in results may be partly explained by the age of the study population.

This study had a few limitations. First, the participants in the present study were middle-aged Koreans, and our results may not be directly applicable to other age groups. Second, we used a single BMI measurement at baseline, which may not account for changes in BMI over follow-ups. Third, although we adjusted for several potential confounders, there may still be residual confounding due to unmeasured factors. Obesity is not only a risk factor for chronic diseases, but also contributes to death as a multifactorial factor, involving genetic, environmental, and behavioral factors that interact in various ways. Although we have assessed the impact of BMI on mortality in this study, there are limitations to fully considering the complex influence of obesity on health.

We examined the impact of BMI on mortality from all causes, cancer, and cardiovascular diseases in a middle-aged Korean population. Our findings suggest that the current WHO BMI cut-off values for Asians could benefit from further evaluation, as they may not fully capture the mortality risks in certain groups.

## Figures and Tables

**Table 1.** Demographic and lifestyle characteristics in the study of body mass index and mortality

Characteristics	Male	Female
Total (n)	39,689	76,272
Age at enrollment (yr)		
Mean±SD	53.7±8.4	52.4±7.7
40-49	13,224 (33.3)	28,629 (37.5)
50-59	14,895 (37.5)	31,818 (41.7)
60-69	11,570 (29.2)	15,825 (20.7)
Education level (yr)		
<12	8,123 (20.5)	27,126 (35.6)
12-16	16,114 (40.6)	32,930 (43.2)
≥16	14,973 (37.7)	15,326 (20.1)
Missing	479 (1.2)	890 (1.2)
Cigarette smoking status		
Never	10,811 (27.2)	73,055 (95.8)
Former	16,331 (41.1)	966 (1.3)
Current	12,413 (31.3)	1,738 (2.3)
Missing	134 (0.3)	513 (0.7)
Alcohol drinking status		
Never	7,895 (19.9)	50,876 (66.7)
Former	2,853 (7.2)	1,462 (1.9)
Current	28,788 (72.5)	23,450 (30.7)
Missing	153 (0.4)	484 (0.6)
Regular exercise		
No	16,736 (42.2)	36,957 (48.5)
Yes	22,799 (57.4)	38,982 (51.1)
Missing	154 (0.4)	333 (0.4)
Menopausal status		
No	-	30,488 (40.0)
Yes	-	45,206 (59.3)
Missing	-	578 (0.8)

Values are presented as number (%). SD, standard deviation.

**Table 2.** Demographic and lifestyle characteristics in the study of waist-to-hip ratio and mortality

Characteristics	Male	Female
Total (n)	39,522	75,982
Age at enrollment (yr)		
Mean±SD	53.7±8.4	52.4±7.7
40-49	13,159 (33.3)	28,522 (37.5)
50-59	14,837 (37.5)	31,694 (41.7)
60-69	11,526 (29.2)	15,766 (20.7)
Education level (yr)		
<12	8,099 (20.5)	27,062 (35.6)
12-16	16,070 (40.7)	32,841 (43.2)
≥16	14,917 (37.7)	15,292 (20.1)
Missing	436 (1.1)	787 (1.0)
Cigarette smoking status		
Never	10,765 (27.2)	72,849 (95.9)
Former	16,284 (41.2)	961 (1.3)
Current	12,367 (31.3)	1,730 (2.3)
Missing	106 (0.3)	442 (0.6)
Alcohol drinking status		
Never	7,864 (19.9)	50,720 (66.8)
Former	2,835 (7.2)	1,449 (1.9)
Current	28,701 (72.6)	23,398 (30.8)
Missing	122 (0.3)	415 (0.5)
Regular exercise		
No	16,685 (42.2)	36,870 (48.5)
Yes	22,725 (57.5)	38,885 (51.2)
Missing	112 (0.3)	227 (0.3)
Menopausal status		
No	-	30,422 (40.0)
Yes	-	45,087 (59.3)
Missing	-	473 (0.6)

Values are presented as number (%). SD, standard deviation.

**Table 3.** Association between body mass index and cause-specific mortality among males and females^1^

Variables	Body mass index (kg/m^2^)						
	<18.5	18.5-<21.0	21.0-<23.0	23.0-<25.0	25.0-<27.0	27.0-<30.0	≥30.0
Male (n)	294	3,645	7,861	11,932	9,494	5,216	1,075
Person-year	2,750	36,283.6	79,163.6	120,892.9	96,231.7	52,703.5	10,717.4
All-cause mortality							
No. of deaths	44	242	390	479	380	215	49
Crude	2.45 (1.89, 3.18)	1.27 (1.08, 1.50)	1.00 (reference)	0.83 (0.72, 0.94)	0.84 (0.73, 0.97)	0.94 (0.79, 1.11)	1.26 (0.93, 1.69)
Adjusted	2.24 (1.73, 2.91)	1.18 (1.00, 1.39)	1.00 (reference)	0.85 (0.74, 0.97)	0.87 (0.75, 1.00)	0.95 (0.81, 1.12)	1.25 (0.92, 1.68)
Cancer mortality							
No. of deaths	16	115	210	269	210	111	33
Crude	1.44 (0.92, 2.26)	1.19 (0.94, 1.50)	1.00 (reference)	0.86 (0.72, 1.03)	0.87 (0.72, 1.05)	0.90 (0.72, 1.13)	1.58 (1.10, 2.29)
Adjusted	1.33 (0.85, 2.08)	1.11 (0.88, 1.40)	1.00 (reference)	0.88 (0.73, 1.05)	0.89 (0.73, 1.08)	0.91 (0.72, 1.15)	1.58 (1.09, 2.28)
Cardiovascular mortality							
No. of deaths	9	45	67	85	73	51	8
Crude	2.53 (1.37, 4.68)	1.39 (0.94, 2.04)	1.00 (reference)	0.85 (0.62, 1.17)	0.93 (0.67, 1.29)	1.29 (0.90, 1.86)	1.16 (0.56, 2.41)
Adjusted	2.27 (1.23, 4.20)	1.26 (0.86, 1.86)	1.00 (reference)	0.87 (0.64, 1.20)	0.96 (0.69, 1.34)	1.33 (0.92, 1.90)	1.16 (0.55, 2.43)
Female (n)	860	12,855	20,726	20,216	12,120	7,045	2,153
Person-year	8,540.5	129,545.7	211,666.3	207,200.2	123,707	71,575.7	21,654.1
All-cause mortality							
No. of deaths	16	169	270	360	230	152	54
Crude	1.51 (1.00, 2.27)	1.25 (1.03, 1.52)	1.00 (reference)	1.13 (0.96, 1.32)	1.06 (0.89, 1.27)	1.15 (0.94, 1.41)	1.42 (1.06, 1.90)
Adjusted	1.47 (0.98, 2.22)	1.23 (1.01, 1.50)	1.00 (reference)	1.12 (0.96, 1.31)	1.05 (0.88, 1.25)	1.11 (0.91, 1.36)	1.33 (0.99, 1.78)
Cancer mortality							
No. of deaths	9	107	172	235	139	81	32
Crude	1.11 (0.62, 2.00)	1.24 (0.97, 1.58)	1.00 (reference)	1.18 (0.97, 1.44)	1.06 (0.84, 1.32)	1.02 (0.78, 1.33)	1.39 (0.96, 2.03)
Adjusted	1.07 (0.59, 1.92)	1.21 (0.95, 1.55)	1.00 (reference)	1.19 (0.98, 1.45)	1.06 (0.85, 1.33)	1.02 (0.78, 1.33)	1.35 (0.92, 1.97)
Cardiovascular mortality							
No. of deaths	4	25	39	60	38	35	8
Crude	2.56 (1.08, 6.06)	1.28 (0.76, 2.14)	1.00 (reference)	1.28 (0.86, 1.91)	1.15 (0.73, 1.80)	1.73 (1.09, 2.73)	1.37 (0.64, 2.94)
Adjusted	2.67 (1.13, 6.33)	1.29 (0.77, 2.16)	1.00 (reference)	1.24 (0.83, 1.85)	1.08 (0.69, 1.70)	1.56 (0.99, 2.48)	1.19 (0.55, 2.55)

Values are presented as hazard ratio (95% confidence interval).

1 The age at recruitment was used as the time scale; Years of education (<12, 12-16, and ≥16 years) and habits of smoking, alcohol consumption (never, former, and current), and regular physical activity (no and yes), and additionally for menopausal status among females (no and yes) were adjusted.

**Table 4.** Association between waist-to-hip ratio and cause-specific mortality among males and females^1^

Variables	Waist-to-hip ratio				
	<0.80	0.80-<0.85	0.85-<0.90	0.90-<0.95	≥0.95
Male (n)	1,714	6,028	14,308	12,929	4,543
Person-year	16,921.9	60,894.8	145,407.6	130,331.1	130,331.1
All-cause mortality					
No. of deaths	73	213	538	655	655
Crude	1.40 (1.09, 1.78)	1.02 (0.87, 1.20)	1.00 (reference)	1.18 (1.05, 1.32)	1.43 (1.24, 1.64)
Adjusted	1.38 (1.08, 1.77)	1.03 (0.88, 1.21)	1.00 (reference)	1.15 (1.02, 1.29)	1.28 (1.11, 1.47)
Cancer mortality					
No. of deaths	36	113	305	358	153
Crude	1.22 (0.87, 1.73)	0.97 (0.78, 1.20)	1.00 (reference)	1.13 (0.97, 1.32)	1.20 (0.99, 1.46)
Adjusted	1.22 (0.86, 1.72)	0.97 (0.78, 1.21)	1.00 (reference)	1.10 (0.94, 1.28)	1.08 (0.88, 1.31)
Cardiovascular mortality					
No. of deaths	16	33	98	125	66
Crude	1.63 (0.96, 2.77)	0.86 (0.58, 1.27)	1.00 (reference)	1.25 (0.96, 1.63)	1.65 (1.21, 2.27)
Adjusted	1.62 (0.95, 2.75)	0.86 (0.58, 1.28)	1.00 (reference)	1.22 (0.93, 1.59)	1.48 (1.08, 2.02)
Female (n)	21,990	22,597	19,764	8,912	2,719
Person-year	223,367.2	231,719.0	201,363.9	89,891.2	27,638.0
All-cause mortality					
No. of deaths	232	326	385	211	97
Crude	0.96 (0.81, 1.14)	0.94 (0.81, 1.09)	1.00 (reference)	1.01 (0.85, 1.19)	1.26 (1.01, 1.58)
Adjusted	0.98 (0.82, 1.16)	0.95 (0.81, 1.10)	1.00 (reference)	0.99 (0.84, 1.17)	1.21 (0.97, 1.51)
Cancer mortality					
No. of deaths	165	208	236	123	43
Crude	1.03 (0.84, 1.26)	0.95 (0.78, 1.14)	1.00 (reference)	0.99 (0.79, 1.23)	0.97 (0.70, 1.34)
Adjusted	1.01 (0.82, 1.24)	0.94 (0.78, 1.13)	1.00 (reference)	0.98 (0.79, 1.22)	0.94 (0.68, 1.31)
Cardiovascular mortality					
No. of deaths	25	55	69	46	14
Crude	0.64 (0.40, 1.02)	0.92 (0.65, 1.32)	1.00 (reference)	1.19 (0.82, 1.73)	0.96 (0.54, 1.70)
Adjusted	0.71 (0.44, 1.14)	0.96 (0.67, 1.37)	1.00 (reference)	1.13 (0.78, 1.65)	0.88 (0.49, 1.57)

Values are presented as hazard ratio (95% confidence interval).

1 The age at recruitment was used as the time scale; Years of education (<12, 12-16, and ≥16 years) and habits of smoking, alcohol consumption (never, former, and current), and regular physical activity (no and yes), and additionally for menopausal status among females (no and yes) were adjusted.
